# The Impact of Cognitive Biases on Professionals’ Decision-Making: A Review of Four Occupational Areas

**DOI:** 10.3389/fpsyg.2021.802439

**Published:** 2022-01-04

**Authors:** Vincent Berthet

**Affiliations:** ^1^Université de Lorraine, 2LPN, Nancy, France; ^2^Psychology and Neuroscience Lab, Centre d’Économie de la Sorbonne, Université de Lorraine, CNRS UMR 8174, Paris, France

**Keywords:** decision-making, cognitive biases, heuristics, management, finance, medicine, law

## Abstract

The author reviewed the research on the impact of cognitive biases on professionals’ decision-making in four occupational areas (management, finance, medicine, and law). Two main findings emerged. First, the literature reviewed shows that a dozen of cognitive biases has an impact on professionals’ decisions in these four areas, overconfidence being the most recurrent bias. Second, the level of evidence supporting the claim that cognitive biases impact professional decision-making differs across the areas covered. Research in finance relied primarily upon secondary data while research in medicine and law relied mainly upon primary data from vignette studies (both levels of evidence are found in management). Two research gaps are highlighted. The first one is a potential lack of ecological validity of the findings from vignette studies, which are numerous. The second is the neglect of individual differences in cognitive biases, which might lead to the false idea that all professionals are susceptible to biases, to the same extent. To address that issue, we suggest that reliable, specific measures of cognitive biases need to be improved or developed.

## Introduction

When making judgments or decisions, people often rely on simplified information processing strategies called heuristics, which may result in systematic, predictable errors called *cognitive biases* (hereafter CB). For instance, people tend to overestimate the accuracy of their judgments (overconfidence bias), to perceive events as being more predictable once they have occurred (hindsight bias), or to seek and interpret evidence in ways that are partial to existing beliefs and expectations (confirmation bias). In fact, the seminal work of Kahneman and Tversky on judgment and decision-making in the 1970s opened up a vast research program on how decision-making deviates from normative standards (e.g., Tversky and Kahneman, 1974; Kahneman et al., 1982; Gilovich et al., 2002).

The “heuristics and biases” program has been remarkably fruitful, leading to unveiling dozens of CB and heuristics in decision-making (e.g., Baron, 2008, listed 53 such biases). While this research turned out to have a large impact in the academic field and beyond (Kahneman, 2011), it is worth noting that it led to some debate (Vranas, 2000; Pohl, 2017). In particular, Gigerenzer (1991, 1996) (Gigerenzer et al., 2008) outlined that Kahneman and Tversky relied upon a narrow view of normative rules (probability theory), leading them to ask participants to make artificial judgments (e.g., estimating the probability of single events) likely to result in so-called “errors.” Gigerenzer also pointed out the overemphasis on decision errors and the lack of theory behind the heuristics-and-biases approach, which eventually results in a list of cognitive errors with no theoretical framework. However, there have been several attempts to overcome this shortcoming, such as the reframing of the heuristics-and-biases literature in terms of the concept of attribute substitution (Kahneman and Frederick, 2002) and the various taxonomies of CB advanced based on dual-process models (e.g., Stanovich et al., 2008).

While early research on CB was conducted on lay participants to investigate decision-making in general, there has been a large interest in how such biases may impede professional decision-making in areas, such as management (e.g., Maule and Hodgkinson, 2002), finance (e.g., Baker and Nofsinger, 2002), medicine (e.g., Blumenthal-Barby and Krieger, 2015), and law (e.g., Rachlinski, 2018). Consider, for example, the framing effect, when making risky decisions, people prefer sure gains over more risky ones, whereas they prefer risky losses over sure ones (Kahneman and Tversky, 1979). Therefore, framing a problem in terms of gains versus losses can significantly impact decision-making. In most lawsuits for instance, plaintiffs choose between a sure gain (the settlement payment) and a potential larger gain (in the case of further litigation) while defendants choose between a sure loss (the settlement payment) and a potential larger loss (in the case of further litigation). In fact, when considering whether the parties should settle the case, judges evaluating the case from the plaintiff’s perspective are more likely to recommend settlement than those evaluating the case from the defendant’s perspective (Guthrie et al., 2001). Likewise, when asking to rate the effectiveness of a drug, presenting the results of a hypothetical clinical trial in terms of absolute survival (gain), absolute mortality (loss), or relative mortality reduction (gain) influences the ratings of doctors (Perneger and Agoritsas, 2011).

For the sake of convenience, we list below the common definition of the main CB considered in this review.

*Anchoring Bias* is the tendency to adjust our judgments (especially numerical judgments) toward the first piece of information (Tversky and Kahneman, 1974).

*Availability bias* is the tendency by which a person evaluates the probability of events by the ease with which relevant instances come to mind (Tversky and Kahneman, 1973).

*Confirmation bias* is the tendency to search for, to interpret, to favor, and to recall information that confirms or supports one’s prior personal beliefs (Nickerson, 1998).

*Disposition effect* is the tendency among investors to sell stock market winners too soon and hold on to losers too long (Shefrin and Statman, 1985). This tendency is typically related to loss aversion (Kahneman and Tversky, 1979).

*Hindsight bias* is a propensity to perceive events as being more predictable, once they have occurred (Fischhoff, 1975).

*Omission bias* is the preference for harm caused by omissions over equal or lesser harm caused by acts (Baron and Ritov, 2004).

*Outcome bias* is the tendency to judge the quality of a decision based on the information about the outcome of that decision. These judgments are erroneous in respect to normative assumption that “information that is available only after decision is made is irrelevant to the quality of the decision” (Baron and Hershey, 1988, p. 569).

*Overconfidence bias* is a common inclination of people to overestimate their own abilities to successfully perform a particular task (Brenner et al., 1996).

*Relative risk bias* is a stronger inclination to choose a particular treatment when presented with the relative risk than when presented with the same information described in terms of the absolute risk (Forrow et al., 1992).

*Susceptibility to framing* is the tendency for people to react differently to a single choice depending on whether it is presented as a loss or a gain (Tversky and Kahneman, 1981).

In the present paper, we review the research on the impact of CB on professional decision-making in four areas: management, finance, medicine, and law. Those applied areas were selected as they have led to the highest number of publications on this topic so far (see “Materials and Methods”). This study aims to address the following research questions:

1. Assess the claim that CB impact professionals’ decision-making
2. Assess the level of evidence reported in the empirical studies
3. Identify the research gaps

We take a narrative approach to synthesizing the key publications and representative empirical studies to answer these research questions. To the best of our knowledge, this study is the first literature review on this research topic covering multiple areas together. This review is narrative, as opposed to a systematic review, which is one of its limitations. However, it aims to be useful both to researchers and professionals working in the areas covered.

The present paper is structured as follows. The Methods section provides details about the methodology used to conduct the literature review. In the following sections, we review the key findings in each of the four occupational areas covered. Finally, in the Discussion section, we answer the three research questions addressed in light of the findings reviewed.

## Materials and Methods

We conducted a systematic literature search using the Web of Science (WoS) database with the search terms “cognitive biases AND decision making.” The search criteria included research articles, review articles, or book chapters with no restriction regarding the time period. We focused on the WoS database as the “Web of Science Categories” filter would offer a practical mean to select the applied areas covered. Admittedly, the results of our review might have been different had we covered more databases; however, as our strategy was to review the key publications and representative empirical studies in each of the areas selected, we reasoned that virtually every database would have led to these records.

**Figure 1:**
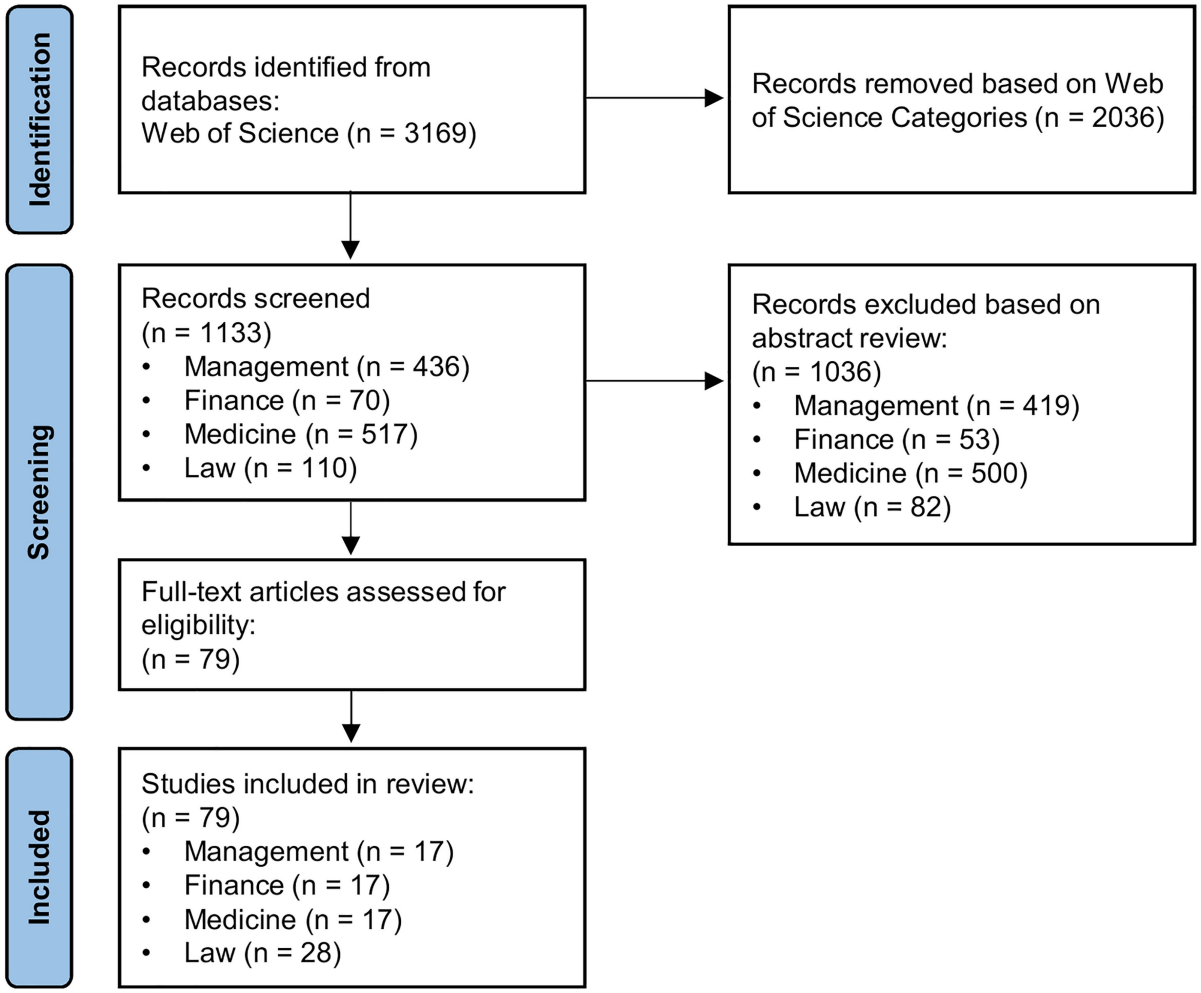
PRISMA flowchart of article search and collection.

The PRISMA flowchart in Figure 1 illustrates the process of article search and selection in this study. The WoS search led to a total of 3,169 records. Before screening, we used the “Web of Science Categories” filter to identify and select the four applied areas with the highest number of publications. Those areas were management (*n* = 436), which merged the categories “Management” (*n* = 260) and “Business” (*n* = 176); medicine (*n* = 517), which merged the categories “Psychiatry” (*n* = 261), “Health Care Sciences Services” (*n* = 112), “Medicine General Internal” (*n* = 94), “Radiology Nuclear Medicine Medical Imaging” (*n* = 22), “Critical Care Medicine” (*n* = 14), and “Emergency Medicine” (*n* = 14); and law (*n* = 110) and finance (*n* = 70). Noteworthy, while the category “Psychology Applied” was associated with a significant number of publications (*n* = 146), a closer examination revealed that the majority of them was related to other applied areas (e.g., management, medicine, law, and ergonomics). Accordingly, this category was not included in the review. The abstracts selected were reviewed according to two inclusion criteria: (1) the article had a clear focus on cognitive biases and decision-making (e.g., not on implicit biases); (2) the article reported a review (narrative or systematic) on the topic or a representative empirical study. This screening led to a selection of 79 eligible articles, which were all included in the review.

## Management

The life of any organization is made of crucial decisions. According to Eisenhardt and Zbaracki (1992, p. 17), strategic decisions are “those infrequent decisions made by the top leaders of an organization that critically affect organizational health and survival.” For instance, when Disney decided to locate Euro Disney in Paris or when Quaker decided to acquire Snapple, these companies took strategic decisions.

A defining feature of strategic decisions is their lack of structure. While other areas of management deal with recurring, routinized, and operationally specific decisions, strategic issues and problems tend to be relatively ambiguous, complex, and surrounded by risk and uncertainty (Hodgkinson, 2001). How do managers actually deal with such decisions? Much of early research on strategic decision-making was based on a neoclassical framework with the idea that strategists in organizations are rational actors. However, the seminal work of Kahneman and Tversky in the 1970s questioned this assumption (Hardman and Harries, 2002). In fact, the very notion of “bounded rationality” emerged in the study of organizations (March and Simon, 1958). One might argue that the issue of individual biases in strategic decision-making is of limited relevance as strategic decisions are the product of organizations rather than individuals within the context of a wider sociopolitical arena (Mintzberg, 1983; Johnson, 1987). However, individual (micro) factors might help explain organizational (macro) phenomena, an idea promoted by behavioral strategy (Powell et al., 2011).

The “heuristics and biases” program revived the interest for bounded rationality in management with the idea that decision-makers may use heuristics to cope with complex and uncertain environments, which in turn may result in inappropriate or suboptimal decisions (e.g., Barnes, 1984; Bazerman, 1998). Indeed, it is relatively easy to see how biases, such as availability, hindsight, or overconfidence, might play out in the strategic decision-making process. For instance, it may seem difficult in hindsight to understand why IBM and Kodak failed to see the potential that Haloid saw (which led to the Xerox company). The hindsight bias can actually lead managers to distort their evaluations of initial decisions and their predictions (Bukszar and Connolly, 1988). Likewise, practicing auditors of major accounting firms are sensitive to anchoring effects (Joyce and Biddle, 1981) and prospective entrepreneurs tend to neglect base rates for business failures (Moore et al., 2007).

To our knowledge, no systematic review of empirical research on the impact of heuristics and CB on strategic decision-making has been published to date. Whereas the idea that CB could affect strategic decisions is widely recognized, the corresponding empirical evidence is quite weak. Most research on this topic consists in narrative papers relying upon documentary sources and anecdotal evidence (e.g., Duhaime and Schwenk, 1985; Lyles and Thomas, 1988; Huff and Schwenk, 1990; Zajac and Bazerman, 1991; Bazerman and Moore, 2008). In fact, the typical paper describes a few CB and provides for each one examples of how a particular bias can lead to poor strategic decisions (see Barnes, 1984, for a representative example). While the examples provided are often compelling, such research faces severe methodological limitations.

The work of Schwenk (1984, 1985) is representative of that type of research. This author identified three different stages of the strategic decision process (goal formulation and problem identification, strategic alternatives generation, evaluation of alternatives, and selection of the best one) and a set of heuristics and biases that might affect decisions at each stage. Schwenk also provided for each bias an illustrative example of how the bias may impede the overall quality of strategic decisions. For example, the representativeness heuristics may affect the stage of evaluation and selection of the alternatives. To illustrate this, Schwenk mentioned the head of an American retail organization (Montgomery Ward) who held a strong belief that there would be a depression at the end of the Second World War as was the case after World War I. Based on this belief, this executive decided not to allow his company to expand to meet competition from his rival (Sears), which led to a permanent loss of market share to Sears. Schwenk (1988) listed ten heuristics and biases of *potential* key significance in the context of strategic decision-making (availability, selective perception, illusory correlation, conservatism, law of small numbers, regression bias, wishful thinking, illusion of control, logical reconstruction, and hindsight bias).

In a similar vein, Das and Teng (1999) proposed a framework to explore the presence of four basic types of CB (prior hypotheses and focusing on limited targets, exposure to limited alternatives, insensitivity to outcome probabilities, and illusion of manageability) under five different modes of decision-making (rational, avoidance, logical incrementalist, political, and garbage can). They proposed that not all basic types of biases are robust across all kinds of decision processes; rather, their selective presence is contingent upon the specific processes that decision makers engage in. For instance, the garbage can mode (Cohen et al., 1972) depicts decision-making processes as organized anarchies, in which a decision is largely dependent on chance and timing. In this kind of process, decision makers do not know their objectives *ex ante*, but merely look around for decisions to make. Das and Teng (1999) hypothesized that managers under the garbage can mode will be exposed to limited alternatives and insensitive to outcome probabilities. On the contrary, managers under the rational mode would be exposed to prior hypotheses and illusion of manageability. This framework, however, is not supported by rigorous empirical evidence.

It is not difficult to list examples of poor strategic decisions that can be readily interpreted – in hindsight – as the result of heuristics and biases. However, the claim that CB influence strategic decisions requires to be tested more directly through laboratory research and experimental studies (Maule and Hodgkinson, 2002). It is worth noting that such research is scarce, probably because of its lack of ecological validity, an issue of primary importance in the field of management research (Schwenk, 1982). Still, two CB in particular have been studied quantitatively, the framing effect and CEO overconfidence.

Hodgkinson et al. (1999) used an experimental setting to investigate the effect of framing on strategic decisions. Following the “Asian Disease” problem (Tversky and Kahneman, 1981), they presented subjects (undergraduate management students) with a 500-word case vignette giving a brief history of a company that manufactured and distributed fast paint-drying systems. A positive and a negative frame were used and participants were asked to adopt the role of a board member facing a major strategic decision and to indicate which of two alternative options they would choose. The positive frame emphasized gains from a reference point of no profit, whereas the negative frame highlighted losses from a reference point where the target profit is achieved (£3 million). In addition, participants were either asked to choose between the presented options directly or to represent the ways in which they thought about the problem in the form of a causal map prior to making their choice. It turned out that when participants made their decisions directly, a massive framing effect was found (45.5% of participants chose the risk-averse option in the positive frame versus 9% in the negative frame). However, no framing effect was observed when participants were asked to draw a causal map before making their choice (36.4% of the participants opted for the risk-averse option in both versions). Interestingly, Hodgkinson et al. reported the same findings on experienced participants (senior managers in a banking organization).

Another CB that led to a large amount of empirical research in strategic management is CEO overconfidence. Overconfidence has various aspects: overprecision, overestimation, and overplacement (Moore and Schatz, 2017). Regarding overprecision, Ben-David et al. (2013) investigated the accuracy of stock market predictions made by senior finance executives (the majority of them being CFOs). The data were collected in 40 quarterly surveys conducted between June 2001 and March 2011. Ben-David et al. asked participants to predict one- and 10-year market-wide stock returns and to provide an 80% confidence interval for their predictions (“Over the next year, I expect the annual SandP 500 return will be: There is a 1-in-10 chance the actual return will be less than ___%; I expect the return to be: ___%; There is a 1-in-10 chance the actual return will be greater than ___%.”). It turned out that the CFOs were severely miscalibrated as: the realized one-year SandP 500 returns fall within their 80% confidence intervals only 36.3% of the time. Even during the least volatile quarters in the sample, only 59% of realized returns fall within the 80% confidence intervals provided. The comparison of the size of the CFOs’ confidence intervals to the distribution of historical one-year returns revealed that their confidence intervals were too narrow. Indeed, CFOs provide an average confidence interval of 14.5%, whereas the difference between the 10th and 90th return percentiles from the realized distribution of the one-year SandP 500 returns is 42.2% (only 3.4% of CFOs provided confidence intervals wider than 42.2%).

Managers also overestimate their abilities, particularly with regard to the illusion of control. In their review on risk perception among managers, March and Shapira (1987) reported that most managers (1) consider that they take risks wisely and that they are less risk-averse than their colleagues, (2) perceive risk as largely controllable, and (3) attribute this controllability to skills and information.

Finally, executives also appear to be overconfident with regard to overplacement. Malmendier and Tate (2005) assessed CEO overconfidence through revealed preferences, examining how they exercised their options. A CEO persistently exercising options later than suggested by the benchmark reveals his belief in his ability to keep the company’s stock price rising and that he or she wants to profit from expected price increases by holding the options. Using panel data on personal portfolio and corporate investment decisions of Forbes 500 CEOs, Malmendier and Tate reported that most of CEO excessively hold company stock options, thereby failing to reduce their personal exposure to company-specific risk. CEO overconfidence is also believed to be involved in merger decisions. As overconfident CEOs overestimate their ability to generate returns, they are supposed to overpay for target companies and undertake value-destroying mergers. Using two measures of CEO overconfidence (CEOs’ personal over-investment in their company and their press portrayal), Malmendier and Tate (2008) provided support for that hypothesis: the odds of making an acquisition are 65% higher if the CEO is classified as overconfident.

## Finance

The case of CB in finance is special. In the 1980s, CB were invoked to account for observations on markets in disagreement with the predictions of standard finance. This paradigm relies upon expected utility theory, assuming that investors make rational decisions under uncertainty (i.e., maximizing utility). Standard finance produced core theoretical concepts, such as arbitrage, portfolio theory, capital asset pricing theory, and efficient market hypothesis, all assuming rational investors. In the 1970s, some observations on financial markets relative to trading behavior, volatility, market returns, and portfolio selection turned out to be inconsistent with the framework of standard finance (“anomalies”). Psychological biases (micro level) were invoked as theoretical explanations of these market anomalies (macro level), launching the field of behavioral finance (Shiller, 2003). In particular, behavioral finance capitalized on prospect theory (Kahneman and Tversky, 1979), a more realistic view of decision-making under uncertainty that expected utility theory. A prime example is how (myopic) loss aversion – a key concept of prospect theory – can account for the equity premium puzzle (i.e., the excessively high difference between equity returns and the return of Treasury bills; Benartzi and Thaler, 1995).

Here, we focus on investment decision-making in individual investors (Shefrin, 2000; Baker and Nofsinger, 2010; Baker and Ricciardi, 2014) and how CB may impede such decisions (see Baker and Nofsinger, 2002, and Kumar and Goyal, 2015, for reviews).^1^ In fact, financial economists have distinguished between two types of investors in the market, arbitrageurs and noise traders. While the latter is assumed to be fully rational, noise traders are investors prone to CB (De Long et al.,1990), which results in under-diversified portfolios. Various CB have been invoked to account for poor individual investment decisions, resulting in suboptimal portfolio management. For example, investors tend to favor stocks that performed well during the past 3–5 years (“winners”) over stocks that performed poorly (“losers”), neglecting that because of regression to the mean, the losers will tend to outperform the winners over the next years (actually by 30%; De Bondt and Thaler, 1985). Investors may exhibit a home bias (an instance of familiarity bias), a tendency to invest the majority of their portfolio in domestic equities rather than diversifying it into foreign equities (Coval and Moskowitz, 1999). Investors may also fall prey to herding, a tendency to follow blindly what other investors do (Grinblatt et al., 1995).

Two CB have been particularly studied in investment decision-making: overconfidence and disposition effect (see the systematic review of Kumar and Goyal, 2015). On the one hand, investors are usually overconfident with regard to the precision of their forecasts. When asked to predict the future return or price of a stock, investors report confidence intervals that are too narrow compared to the actual variability of prices (e.g., De Bondt, 1998). Investors also overestimate their ability to beat the market. Baker and Nofsinger (2002) reported a finding of a Gallup survey in 2001 revealing that on average, investors estimated that the stock market return during the next 12 months would be 10.3% while estimating that their portfolio return would be 11.7%. Barber and Odean (2001) reported evidence that overconfidence in investors is related to gender. Based on a sample of 35,000 individual accounts over a six-year period, their findings showed that males exhibit more overconfidence regarding their investing abilities and also trade more often than females. Overconfidence in investors makes them more prone to take high risks (Chuang and Lee, 2006) and trade too much (Odean, 1999; Statman et al., 2006; Glaser and Weber, 2007), which results in poor financial performance (consequent transaction costs and losses). For instance, trading turnover and portfolio returns are negatively correlated: of 66,465 households with accounts at a large discount broker during 1991–1996, households that trade most had an annual return of 11.4% while the average annual return was 16.4% (Barber and Odean, 2000).

On the other hand, the disposition effect is the tendency by which investors tend to sell winning stocks too early while holding on to losing positions for too long (Shefrin and Statman, 1985). Based on trading records for 10,000 accounts at a large discount brokerage house, Odean (1998) reported that on average, winning investments are 50% more likely to be sold than losing investment (similar results were obtained in other countries, such as France; Boolell-Gunesh et al., 2009). The disposition effect originates in loss aversion described by prospect theory (Kahneman and Tversky, 1979).

## Medicine

The idea that cognitive failures are a primary source of medical errors has become prevalent in the medical literature (e.g., Detmer et al., 1978; Dawson and Arkes, 1987; Schmitt and Elstein, 1988; Elstein, 1999; Croskerry, 2003; Klein, 2005). In fact, emergency medicine has been described as a “natural laboratory of error” (Bogner, 1994). Among medical errors, diagnostic errors have received particular attention (Graber, 2013). Indeed, there is increasing evidence that mental shortcuts during information processing contribute to diagnostic errors (e.g., Schnapp et al., 2018).

It is not difficult to see how CB may impact medical decisions. Blumenthal-Barby and Krieger (2015) provided the following examples. A parent might refuse to vaccinate her child after she sees a media report of a child who developed autism after being vaccinated (availability bias). A patient with atrial fibrillation might refuse to take warfarin because she is concerned about causing a hemorrhagic stroke despite greater risk of having an ischemic stroke if she does not take warfarin (omission bias). Indeed, early papers on this topic were primarily narrative reviews suggesting a *possible impact* of CB on medical decision-making. These papers follow the same logic: they first provide a general description of a couple of CB and then describe how these shortcuts can lead physicians to make poor decisions, such as wrong diagnoses (e.g., Dawson and Arkes, 1987; Elstein, 1999; Redelmeier, 2005). But narrative reviews provide limited evidence. As Zwaan et al. (2017, p.105) outlined, “While these papers make a formidable argument that the biases described in the literature *might* cause a diagnostic error, empirical evidence that any of these biases actually causes diagnostic errors is sparse.”

On the other hand, studies that investigated the *actual* impact of CB on medical decisions are mainly experimental studies using written cases (hypothetical vignettes) designed to elicit a particular bias. A typical example of vignette study is that of Mamede et al. (2010) on the effect of availability bias on diagnostic accuracy. In a first phase, participants (first-year and second-year internal medicine residents) were provided with 6 different cases and they were asked to rate the likelihood that the indicated diagnosis was correct (all cases were based on real patients with a confirmed diagnosis). Then, participants were asked to diagnose 8 new cases as quickly as possible, that is, relying on non-analytical reasoning. Half of those new cases were similar to the cases encountered in phase 1, so that the availability bias was expected to reduce diagnostic accuracy for those four cases. Second-year residents had actually lower diagnostic accuracy on cases similar to those encountered in phase 1 as compared to other cases, as they provided the phase 1 diagnosis more frequently for phase 2 cases they had previously encountered than for those they had not.

While vignette-based studies are the most frequent, researchers in this area have used diverse strategies (Blumenthal-Barby and Krieger, 2015). For instance, Crowley et al. (2013) developed a computer-based method to detect heuristics and biases in diagnostic reasoning as pathologists examine virtual slide cases. Each heuristic or bias is defined as a particular sequence of hypothesis, findings, and diagnosis formulation in the diagnostic reasoning interface (e.g., availability bias is considered to occur if in a sequence of three cases where the third case has a different diagnosis than the two previous ones, the participant makes an incorrect diagnosis in the third case such that the diagnosis is identical to the correct diagnosis in the two immediately preceding cases). Such a procedure allows for examining the relationships between heuristics and biases, and diagnostic errors.

Another methodology consists in reviewing instances where errors occurred, to which CB presumably contributed (e.g., Graber et al., 2005). However, studies following this methodology are vulnerable to hindsight bias: since reviewers are aware that an error was committed, they are prone to identify biases *ex post* (Wears and Nemeth, 2007). The fact that bias can be in the eye of the beholder has been supported by Zwaan et al. (2017) who asked 37 physicians to read eight cases and list which CB were present from a list provided. In half the cases, the outcome implied a correct diagnosis; in the other half, it implied an incorrect diagnosis. Physicians identified more biases when the case outcome implied an incorrect diagnosis (3.45 on average) than when it implied a correct one (1.75 on average).

To date, two systematic reviews have been published on the impact of CB on medical decision-making. Reviewing a total of 213 studies, Blumenthal-Barby and Krieger (2015) reported the following findings: (1) 77% of the studies (*N* = 164) were based on hypothetical vignettes; (2) 34% of studies (*N* = 73) investigated medical personnel; (3) 82% of the studies (*N* = 175) were conducted with representative populations; (4) 68% of the studies (*N* = 145 studies) confirmed a bias or heuristic in the study population; (5) the most studied CB are loss/gain framing bias (72 studies, 24.08%), omission bias (18 studies, 6.02%), relative risk bias (29 studies, 9.70%), and availability bias (22 studies, 7.36%); (6) the results regarding loss/gain framing bias are mixed with 39% of studies (*N* = 28) confirming an effect, 39% (*N* = 28) confirming an effect only in a subpopulation, and 22% (*N* = 16) disconfirming any effect; (7) 25 of 29 studies (86%) supported the impact of relative risk bias on medical decisions; and (8) 14 of 18 studies (78%) supported the impact of omission bias on medical decisions.

Saposnik et al. (2016) conducted a similar review but including only 20 studies. These authors reported that as: (1) 60% of the studies (*N* = 12) targeted CB in diagnostic tasks; (2) framing effect (*N* = 5) and overconfidence (*N* = 5) were the most common CB while tolerance to risk or ambiguity was the most commonly studied personality trait (*N* = 5); and (3) given that the large majority of the studies (85%) targeted only one or two biases, the true prevalence of CB influencing medical decisions remains unknown. Moreover, there was a wide variability in the reported prevalence of CB. For example, when analyzing the three most comprehensive studies that accounted for several CB (Ogdie et al., 2012; Stiegler and Ruskin, 2012; Crowley et al., 2013), it turned out that the availability bias ranged from 7.8 to 75.6% and anchoring bias from 5.9 to 87.8%; (4) the presence of CB was associated with diagnostic inaccuracies in 36.5 to 77% of case-scenarios. Physicians’ overconfidence, anchoring effect, and information or availability bias may be associated with diagnostic inaccuracies; (5) only seven studies (35%) provided information to evaluate the association between physicians’ CB and therapeutic or management errors. Five of these studies (71.4%) showed an association between CB (anchoring, information bias, overconfidence, premature closure, representativeness, and confirmation bias) and therapeutic or management errors.

## Justice

Based on the legal realism’ premise that “judges are human,” the recent years have seen a growing interest for judicial decision-making (e.g., Klein and Mitchell, 2010; Dhami and Belton, 2017; Rachlinski, 2018). This topic covers issues, such as cognitive models of judicial decision-making (e.g., the story model), the impact of extralegal factors on decisions, prejudice (e.g., gender bias and racial bias), moral judgments, group decision-making, or the comparison of lay and professional judges. It is worth noting that most research on judicial decision-making has focused on how jurors decide cases, relying on jury simulations (MacCoun, 1989). Here, we focus on how professional judges might be prone to CB. One might easily consider how CB could hamper judicial decisions. In a narrative fashion, Peer and Gamliel (2013) reviewed how such biases could intervene during the hearing process (confirmation bias and hindsight bias), ruling (inability to ignore inadmissible evidence), and sentencing (anchoring effects). In fact, research suggests that judges, prosecutors, and other professionals in the legal field might rely on heuristics to produce their decisions, which leaves room for CB (e.g., Guthrie et al., 2007; Helm et al., 2016; Rachlinski and Wistrich, 2017).^2^

Researchers investigating judges’ decision-making have mainly relied upon archival studies (document analyses of court records) and experimental studies in which judges are asked to decide on hypothetical cases. In archival studies, researchers examine if judges’ decisions in actual cases exhibit features of irrationality. For instance, Ebbesen and Konecni (1975) investigated which information felony court judges considered when deciding the amount of bail to set. When presented with fictitious cases, the judges’ decisions were influenced by relevant information, such as prior criminal record, but their actual bail decisions relied almost exclusively on prosecutorial recommendations. That is, judges seem to be (too) heavily affected by prosecutors’ recommendations. Another example of archival study is the infamous research of Danziger et al. (2011) who highlighted a cycle in repeated judicial rulings: judges are initially lenient, then progressively rule more in favor of the status quo over time, and become lenient again after a food break. This would suggest that psychological factors, such as mental fatigue, could influence legal decisions (but see Weinshall-Margel and Shapard, 2011). Archival studies, however, are limited by the difficulty to control for unobserved variables.

On the other hand, vignette studies consist in presenting judges with hypothetical scenarios simulating real legal cases. As in the medical field, researchers have primarily relied on such studies. A representative study is that of Guthrie et al. (2001) who administered a survey to 167 federal magistrate judges in order to assess the impact of five CB (anchoring, framing, hindsight bias, inverse fallacy, and egocentric bias) on their decisions regarding litigation problems (see Guthrie et al., 2002, for a summary of the research). Using materials adapting classic cognitive problems into legal ones, Guthrie et al. (2001) reported that judges fell prey to these biases but to various extent. For instance, in order to assess whether judges were susceptible to hindsight bias, Guthrie et al. (2001) presented them with a hypothetical case in which the plaintiff appealed the district court’s decision and asked them to indicate which of three possible outcomes of the appeal was most likely to have occurred. Crucially, they also provided them with the actual outcome of the court of appeals. The outcome significantly influenced judges’ assessments: those informed of a particular outcome were more likely to have identified that outcome as the most likely to have occurred.

In particular, numerous studies have investigated the impact of anchoring effects on judicial decisions (see Bystranowski et al., 2021, for a recent meta-analysis). Judges and jurors are often required to translate qualitative judgments into quantitative decisions (Hans and Reyna, 2011; Rachlinski et al., 2015). While their qualitative judgments on matters, such as the severity of the plaintiff’s injury or the appropriate severity of punishment, show a high degree of consistence and predictability (Wissler et al., 1999), a great amount of variability appears (especially for non-economic and punitive damages) when these qualitative judgments are translated into numbers (e.g., civil damage awards and criminal sentences; Hart et al., 1997; Diamond et al., 1998). This might be explained by the fact that numerical assessments can be prone to anchoring. Facing uncertainty about the amount to determine, judges and especially juries (due to their lack of experience and information about standard practice) tend to rely on any numerical point of reference and make their judgment through adjustments from that number. As these adjustments are often insufficient, the judgments are biased toward the anchor (see Kahneman et al., 1998, for a model describing how individual jurors set punitive damages and the role of anchoring in that process).

Accordingly, numerical values, such as a damage cap (e.g., Hinsz and Indahl, 1995; Robbennolt and Studebaker, 1999), the amount of damages claimed by the plaintiff (Chapman and Bornstein, 1996), the amount of economic damage (Eisenberg et al., 1997, 2006), the sentence imposed in the preceding case, a sentence urged by the prosecutor, or a sentence recommended by a probation officer, might act as anchors in the courtroom, moving the judges’ decisions toward them. Guthrie et al. (2001) reported that in a personal injury suit, an irrelevant factor, such as a number in a pre-trial motion (used to determine whether the damages met the minimum limit for federal court), could act as an anchor. They presented judges with a description of a serious personal injury suit in which only damages were at issue and asked them to estimate how much they would award the plaintiff in compensatory damages. Prior to this estimation, half of the judges were asked to rule on a pre-trial motion filed by the defendant to have the case dismissed for failing to meet the jurisdictional minimum in a diversity suit ($75,000). It turned out that the judges who were asked only to determine the damage award provided an average estimate of $1,249,000 while the judges who first ruled on the motion provided an average estimate of $882,000.

Enough and Mussweiler (2001) conducted a series of research on how recommendations anchor judicial decisions, even when they are misleading. In their 2001 paper, they showed that sentencing decisions tend to follow the sentence demanded by the prosecutor. When told that the prosecutor recommended a sentence of 34 months, criminal trial judges recommended on average 8 months longer in prison (*M* = 24.41 months) than when told that the sentence should be 12 months (*M* = 17.64) for the same crime. This anchoring effect was independent of the perceived relevance of the sentencing demand, and judges’ experience. Englich et al. (2006) reported that anchoring even occurs when the sentencing demand is determined randomly (the result of a dice throw). Interestingly, Englich et al. (2005) found that the defense’s sentencing recommendation is actually anchored on the prosecutor’s demand, so that the former mediates the impact of the latter on the judge’s decision. Therefore, while it is supposed to be at their advantage, the fact that defense attorneys present their sentencing recommendation after the prosecution might be a hidden disadvantage for the defense.

Along with anchoring, the impact of hindsight bias in the courtroom has been also well documented, mainly in liability cases (Harley, 2007; Oeberst and Goeckenjan, 2016). When determining liability or negligence, judges and juries must assess whether the defendant is liable for a negative outcome (damage or injury). The difficulty is that jurors accomplish this task in retrospect: having knowledge of the outcome, jurors tend to perceive it as foreseeable and accordingly rate highly the negligence or liability of the defendant (Rachlinski et al., 2011). To avoid this bias, the law requires jurors to ignore the outcome information while evaluating the extent to which it should have been foreseen by the defendant. However, research suggests that jurors tend to fall prey to hindsight bias as much as lay persons. When evaluating the precautions took by a municipality to protect a riparian property owner from flood damage, participants assessing the situation in foresight concluded that a flood was too unlikely to justify further precautions. However, participants assessing the situation in hindsight considered that such a decision was negligent and also gave higher estimates for the probability of the disaster occurring (Kamin and Rachlinski, 1995).

Outcome information has been shown to affect jurors’ decisions about punitive damage awards (Hastie et al., 1999) and their decisions about the legality of a search (Casper et al., 1989). In addition, more severe outcomes tend to produce a larger hindsight bias, a result particularly stressed in medical malpractice litigation (LaBine and LaBine, 1996). While the assessment of negligence of the accused physician should be based on his course of action regardless of the outcome, jurors are highly influenced by the severity of a negative medical outcome when determining negligence in medical malpractice cases (Berlin and Hendrix, 1998). Cheney et al. (1989) reviewed 1,004 cases of anesthesia-related negligence and reported that the court had imposed liability on the defendant in over 40 percent of the cases, even though the physician acted appropriately.

There is also significant evidence that confirmation bias (Nickerson, 1998) may impact professional judges’ decisions. In the legal field, confirmation bias has been primarily studied with regard to criminal investigations (Findley and Scott, 2006). Once they become convinced that the suspect is guilty, professionals involved in criminal proceedings (e.g., police officers and judges) may engage in guilt-confirming investigation endeavors (or tunnel vision) by which they undermine alternative scenarios in which the suspect is actually innocent. Several studies reported evidence of confirmation bias in criminal cases. For instance, O’Brien (2009) found that participants (College students) who articulated a hypothesis regarding the suspect early in their review of a mock police file showed bias in seeking and interpreting evidence to favor that hypothesis, thereby demonstrating a case-building mentality against a chosen suspect. Similarly, Lidén et al. (2019) showed that judges’ detentions of suspects trigger a confirmation bias that influences their assessment of guilt and that this bias is affected by who decided about detention. In fact, judges perceived the detained defendants’ statements as less trustworthy and were also more likely to convict when they themselves had previously detained the suspect as compared to when a colleague had decided to detain.^3^

Table 1 provides a summary of the main CB in the four occupational areas reviewed and the corresponding evidence.

**Table 1:** Summary of the main cognitive biases studied in the fields of management, finance, medicine, and law, and corresponding evidence.

	Studies included in the review
Management	Barnes (1984) (multiple, narrative); Ben-David et al. (2013) (overconfidence, empirical); Bukszar and Connolly (1988) (hindsight bias, empirical); Das and Teng (1999) (multiple, theoretical); Duhaime and Schwenk (1985) (multiple, theoretical); Hodgkinson et al. (1999) (framing effect, empirical); Huff and Schwenk (1990) (multiple, theoretical); Joyce and Biddle (1981) (anchoring effect, empirical); Lyles and Thomas (1988) (multiple, theoretical); Malmendier and Tate (2005) (overconfidence, empirical); Malmendier and Tate (2008) (overconfidence, empirical); Maule and Hodgkinson (2002) (multiple, narrative); Moore et al. (2007) (overconfidence, empirical); Schwenk (1988) (multiple, theoretical); Schwenk (1984) (multiple, narrative); Schwenk (1985) (multiple, narrative); Zajac and Bazerman (1991) (blind spot bias, theoretical)
Finance	Baker and Nofsinger (2002) (multiple, review); Barber and Odean (2000) (overconfidence, empirical); Barber and Odean (2001) (overconfidence, empirical); Benartzi and Thaler (1995) (loss aversion, empirical); Boolell-Gunesh et al. (2009) (disposition effect, empirical); Chuang and Lee (2006) (overconfidence, empirical); Coval and Moskowitz (1999) (home bias, empirical); De Bondt and Thaler (1985) (regression to the mean, empirical); De Bondt (1998) (multiple, review); Glaser and Weber (2007) (overconfidence, empirical); Grinblatt et al. (1995) (herding behavior, empirical); Kumar and Goyal (2015) (multiple, review); Odean (1998) (disposition effect, empirical); Odean (1999) (overconfidence, empirical); Shefrin and Statman (1985) (disposition effect, empirical); Shiller (2003) (multiple, narrative); Statman et al. (2006) (overconfidence, empirical)
Medicine	Blumenthal-Barby and Krieger (2015) (multiple, review); Croskerry (2003) (multiple, narrative); Crowley et al. (2013) (multiple, empirical); Dawson and Arkes (1987) (multiple, narrative); Detmer et al. (1978) (multiple, narrative); Elstein (1999) (multiple, narrative); Graber et al. (2005) (multiple, empirical); Klein (2005) (multiple, narrative); Mamede et al. (2010) (availability bias, empirical); Ogdie et al. (2012) (multiple, empirical); Redelmeier (2005) (multiple, narrative); Saposnik et al. (2016) (multiple, review); Schmitt and Elstein (1988) (multiple, narrative); Schnapp et al. (2018) (multiple, empirical); Stiegler and Ruskin (2012) (multiple, review); Wears and Nemeth (2007) (hindsight bias, narrative); Zwaan et al. (2017) (multiple, empirical)
Law	Berlin and Hendrix (1998) (hindsight bias, narrative); Bystranowski et al. (2021) (anchoring effect, review); Casper et al. (1989) (hindsight bias, empirical); Chapman and Bornstein (1996) (anchoring effect, empirical); Cheney et al. (1989) (hindsight bias, empirical); Englich et al. (2005) (anchoring effect, empirical); Englich et al. (2006) (anchoring effect, empirical); Enough and Mussweiler (2001) (anchoring effect, empirical); Findley and Scott (2006) (confirmation bias, theoretical); Guthrie et al. (2001) (multiple, empirical); Guthrie et al. (2007) (multiple, empirical); Guthrie et al. (2002) (multiple, narrative); Harley (2007) (hindsight bias, review); Hastie et al. (1999) (anchoring effect, empirical); Helm et al. (2016) (multiple, empirical); Hinsz and Indahl (1995) (anchoring effect, empirical); Kamin and Rachlinski (1995) (hindsight bias, empirical); LaBine and LaBine (1996) (hindsight bias, empirical); Lidén et al. (2019) (confirmation bias, empirical); O’Brien (2009) (confirmation bias, empirical); Oeberst and Goeckenjan (2016) (hindsight bias, review); Peer and Gamliel (2013) (multiple, narrative); Rachlinski and Wistrich (2017) (multiple, narrative); Rachlinski (2018) (framing effect, empirical); Rachlinski et al. (2011) (hindsight bias, empirical); Rachlinski et al. (2015) (anchoring effect, empirical); Robbennolt and Studebaker (1999) (anchoring effect, empirical)

*After each study, we indicate in brackets the cognitive bias(es) addressed in the study and the article type (narrative, empirical, theoretical, and review)*.

## Discussion

The goal of the present paper was to provide an overview of the impact of CB on professional decision-making in various occupational areas (management, finance, medicine, and law). In all of them, there has been tremendous interest in that issue as revealed by a vast amount of research. Our review provided significant answers to the three research questions addressed.

First, the literature reviewed shows that, overall, professionals in the four areas covered are prone to CB. In management, there is evidence that risky-choice (loss/gain) framing effects and overconfidence (among CEOs) impact decision-making. In finance, there is strong evidence that overconfidence and the disposition effect (a consequence of loss aversion) impact individual investors’ decision-making. Regarding medical decision-making, the systematic review of Blumenthal-Barby and Krieger (2015) revealed that (1) 90% of the 213 studies reviewed confirmed a bias or heuristic in the study population or in a subpopulation of the study; (2) there is strong evidence that omission bias, relative risk bias, and availability bias have an impact on medical decisions, and mixed evidence for the risky-choice framing effect. On the other hand, the systematic review of Saposnik et al. (2016) – based on 20 studies only – reported that physicians’ overconfidence, anchoring, and availability bias were associated with diagnostic errors. Finally, the effects of anchoring, hindsight bias, and confirmation bias on judicial decision-making are well documented. Overall, overconfidence appears as the most recurrent CB over the four areas covered.

Second, the level of evidence supporting the claim that CB impact professionals’ decision-making differs across the four areas covered. In medicine and law, this issue has been primarily evidenced in vignettes studies. Such primary data provide a relevant assessment of CB in decision-making but they face the issue of ecological validity (see below). Accordingly, a mid-level of evidence can be assigned to these findings. On the other hand, following the method of revealed preference by which the preferences of individuals are uncovered through the analysis of their choices in real-life settings, the impact of CB on financial decision-making has been evidenced through secondary data (e.g., trading records), indicating a higher level of evidence. In management, both levels of evidence are found (framing effects were demonstrated in vignette studies while CEO overconfidence was evidenced through secondary data).

A practical implication of these findings is the need for professionals to consider concrete, practical ways of mitigating the impact of CB on decision-making. In finance, this issue has been tackled with programs that aimed to improve financial literacy (Lusardi and Mitchell, 2014). In medicine, debiasing has been considered as a way to reduce the effects of CB (Graber et al., 2002, 2012; Croskerry, 2003; Croskerry et al., 2013). In fact, recent research has reported evidence that the debiasing of decisions can be effective (Morewedge et al., 2015; Sellier et al., 2019). However, a preliminary step to considering practical means of mitigating the impact of CB is to acknowledge this diagnosis. In fact, professionals are reluctant to accept the idea that their decisions may be biased (e.g., Kukucka et al., 2017). Judges, for instance, tend to dismiss the evidence showing the impact of CB on judicial decisions, arguing that most studies did not investigate decisions on real cases (Dhami and Belton, 2017).

Thirdly, our review highlights two major research gaps. The first one is a potential lack of ecological validity of the findings from vignette studies, which are numerous (Blumenthal-Barby and Krieger, 2015). Consider for instance a study designed to test whether sentencing decisions could be anchored by certain information, such as the sentence demanded by the prosecutor (Enough and Mussweiler, 2001). A typical study consists in presenting judges with a vignette describing a hypothetical criminal case and asking them to sentence the defendant (e.g., Rachlinski et al., 2015). If a statistically significant difference is observed between the different anchor conditions, it is concluded that anchoring impacts judges’ sentencing decisions. Does such a finding mean that judges’ sentencing decisions in real cases are affected by anchoring too? Likewise, it has been reported that 90% of judges solve the Wason task incorrectly (Rachlinski et al., 2013) but this does not imply *per se* that confirmation bias impedes judges’ decisions in their regular work. Addressing that issue requires to use more ecological settings, such as mock trials in the case of judicial decision-making (Diamond, 1997).

The second research gap is the neglect of individual differences in CB. This limit was found in the four areas covered. Individual differences have been neglected in decision-making research in general (Stanovich et al., 2011; Mohammed and Schwall, 2012). Indeed, most of the current knowledge about the impact of CB on decision-making relies upon experimental research and group comparisons (Gilovich et al., 2002). For instance, based on the experimental result described above, one might wrongly infer that all judges are susceptible to anchoring, to the same extent. That is why Guthrie et al. (2007, p. 28) clarified that “the fact that we generally observed statistically significant differences between the control group judges and experimental group judges does not mean that every judge made intuitive decisions. […] Our results only show that, as a group, the judges were heavily influenced by their intuition – they do not tell us which judges were influenced and by how much.” In fact, there is clear evidence for individual differences in susceptibility to CB (e.g., Bruine de Bruin et al., 2007).

The issue of individual differences is of primary importance when considering CB in decision-making, especially among professionals. In finance for example, the measurement of the disposition effect at the individual level revealed significant individual differences, 20% of investors showing no disposition effect or a reverse effect (Talpsepp, 2011). Taking full account of individual differences is crucial when considering public interventions aiming to mitigate individual biases: any single intervention might work on individuals highly susceptible to the bias addressed while having no or even harmful effects on individuals moderately susceptible to it (Rachlinski, 2006).

Addressing the issue of individual differences in bias susceptibility requires having standardized, reliable measures (Berthet, 2021). While reliable measures of a dozen CB are currently available, measures of key biases are still lacking (e.g., confirmation bias and availability bias). Most importantly, these measures are generic, using non-contextualized items. Such measures are relevant for research with the purpose of describing general aspects of decision-making (Parker and Fischhoff, 2005; Bruine de Bruin et al., 2007). However, research on individual differences in professional decision-making requires specific measures which items are adapted to the context in which a particular decision is made (e.g., diagnostic decision and sentencing decision). An example is the inventory of cognitive biases in medicine (Hershberger et al., 1994) which aims to measure 10 CB in doctors (e.g., insensitivity to prior probability and insensitivity to sample size) through 22 medical scenarios. The development of such instruments in the context of management, finance, and law is an important avenue for future research on professional decision-making.

## Author Contributions

The author confirms being the sole contributor of this work and has approved it for publication.

## Conflict of Interest

The author declares that the research was conducted in the absence of any commercial or financial relationships that could be construed as a potential conflict of interest.

## Publisher’s Note

All claims expressed in this article are solely those of the authors and do not necessarily represent those of their affiliated organizations, or those of the publisher, the editors and the reviewers. Any product that may be evaluated in this article, or claim that may be made by its manufacturer, is not guaranteed or endorsed by the publisher.

## Footnotes

^1^It should be noted that most research in behavioral finance has focused on individual rather than professional investors (e.g., mutual funds, hedge funds, pension funds, and investment advisors). Findings suggest that institutional investors are prone to various CB but to a lesser extent than individual investors (e.g., Kaustia et al., 2008).

^2^Interestingly, the notion of cognitive bias might also shed light on certain rules of law. For example, Guthrie et al. (2001) presented judges with a problem based on the classic English case Byrne v. Boadle (1863) asked them to assess the likelihood that a warehouse was negligent for an accident involving a barrel that injured a bystander. The materials indicated that when the warehouse is careful, accidents occur one time in 1,000, but that when the warehouse is negligent, accidents occur 90% of the time. The materials also indicated that the defendant is negligent only 1% of the time. Judges overestimated the probability that the defendant was negligent, failing to consider the base rate of negligence. Interestingly, this logical fallacy is implemented in the doctrine (res ipsa loquitur), which instructs judges to take no account of the base rates (Kaye, 1979).

^3^Note that other CB, such as framing and omission bias, might also shed light on judicial decision-making (Rachlinski, 2018). In fact, judges decide cases differently depending on whether the underlying facts are presented as gains or losses (Rachlinski and Wistrich, 2018). Moreover, viewing the acceptance of a claim as the path of action and its dismissal as the path of inaction, omission bias might explain why the acceptance threshold of judges of a plaintiff’s claim is particularly high (Zamir and Ritov, 2012). However, those biases have been much less studied than anchoring and hindsight bias.
